# Prevalence and Risk Factors of Delirium in Patients Admitted to Intensive Care Units: A Multicentric Cross-Sectional Study

**DOI:** 10.7759/cureus.44827

**Published:** 2023-09-07

**Authors:** Fnu Sadaf, Muhammad Saqib, Muhammad Iftikhar, Afaq Ahmad

**Affiliations:** 1 Department of Primary and Secondary Healthcare, Basic Healthcare Unit, Verpal Chattha, Gujranwala, PAK; 2 Department of Internal Medicine, Khyber Teaching Hospital, Peshawar, PAK

**Keywords:** delirium, intensive care units, pakistan, prevalence, critical illness

## Abstract

Background

Delirium is a common and serious complication among critically ill patients in the intensive care unit. Knowledge of the risk factors of delirium can help tremendously in the diagnosis of delirium.

Methods

In April of 2023, a cross-sectional multicenter study was conducted in eight intensive care units (ICUs) across Pakistan. Delirium was assessed using the intensive care delirium screening checklist. Demographic and clinical data were collected, and multivariable logistic regression analysis was performed to identify predictors of delirium. A total of 256 patients were enrolled in the study.

Results

The mean age of participants was 56 (S.D. 12) years. The point prevalence of delirium was 39%, and the point prevalence did not vary significantly among the participating intensive care units. Advanced age, higher Acute Physiology and Chronic Health Evaluation - IV (APACHE IV) scores, and higher Richmond Agitation-Sedation Scale (RASS) scores at enrollment were identified as significant predictors of delirium.

Conclusion

The high prevalence of delirium, observed at 39.0%, emphasizes the importance of proactive screening and effective management strategies in the ICU setting. Healthcare providers in Pakistan should be mindful of these risk factors and implement preventive measures to minimize the occurrence of delirium in critically ill patients. Further research and implementation of targeted interventions are warranted to improve patient outcomes and enhance the overall quality of care in this population.

## Introduction

Delirium is a frequently occurring neurological disorder that is usually characterized by temporary fluctuations in attention, confusion, and disordered thinking. This definition comes from the fifth edition of the Diagnostic and Statistical Manual of Mental Disorders (DSM-5), published by the American Psychiatric Association [1]. Typically, delirium arises in approximately 20-50% of patients who are critically ill and not receiving mechanical ventilation, and in 50-80% of patients who are mechanically ventilated [2-7]. The reported rates of delirium can vary significantly based on the characteristics of the population studied and the diagnostic instruments utilized. Patients who are critically ill and experience delirium are more likely to face a range of adverse outcomes, such as an elevated risk of rehospitalization, lengthier stays in the intensive care unit (ICU) and on mechanical ventilation, higher mortality rates, and increased odds of being discharged to a long-term care facility [5-9]. In addition, delirium has often been associated with higher hospital expenses and long-term impairment of cognitive abilities that can last for several months or even years following discharge [10-15].

The likelihood of experiencing delirium is reliant on the existence of predisposing and precipitating factors such as older individuals who exhibit dementia, functional limitations, and hearing difficulties, as well as those with a previous diagnosis of dementia, cerebrovascular conditions, or seizure disorders [16]. There has been significant variation noted among published studies with respect to various risk factors for delirium. However, the available evidence strongly indicates that both modifiable and non-modifiable risk factors are associated with the occurrence of delirium. These risk factors include the use of benzodiazepines, blood transfusions, dementia, previous coma, advancing age, emergency surgery or trauma before admission to the ICU, and an elevated acute physiology and chronic health evaluation (APACHE) score [17, 18].

The fluctuating nature of delirium, coupled with the lack of regular formal assessments in the ICU, has resulted in its under-recognition, with estimates suggesting that between 30% and 75% of cases go undetected [19, 20]. Given the negative consequences of delirium, the Society of Critical Care Medicine has released Guidelines for the Prevention and Management of Pain, Agitation/Sedation, Delirium, Immobility, and Sleep Disruption in Adult Patients in the ICU, which recommend routine delirium screening using either the Confusion Assessment Method-ICU (CAM-ICU) or the Intensive Care Delirium Screening Checklist (ICDSC) [17]. The two screening tools have been found to have a good overall agreement, with a kappa coefficient of 0.80 (confidence interval (CI) 95% 0.78-0.84; p < 0.001) [21, 22].

The Confusion Assessment Method-ICU (CAM-ICU) is designed to evaluate four key features: the sudden onset of symptoms, fluctuations in symptoms, inattention, and either disorganized thinking or a change in consciousness. This algorithmic approach is focused on assessing the patient and takes between two to five minutes to complete [3]. On the other hand, the ICDSC assesses eight domains, including altered level of consciousness, inattention, disorientation, and psychosis, using a focused patient assessment method. It also considers four additional domains: psychomotor activity, inappropriate speech, sleep disturbance, and fluctuation over the prior and current nursing shift. A score of 4 or higher on the ICDSC is highly indicative of a formal psychiatric diagnosis of delirium [23-25]. The assessment of sedation and agitation in critically ill patients often involves the use of the Richmond Agitation-Sedation Scale (RASS), which is often employed in conjunction with the CAM-ICU or ICDSC for detecting delirium [26, 27].

Studies that investigate the prevalence of delirium and associated risk factors among critically ill patients in Pakistan are limited in number. Only a few reports have been published thus far. For instance, a survey conducted in one Pakistani ICU showed that an incidence rate of 21.8% delirium was observed [28]. Another group of investigators found the prevalence of delirium to be 22% [29]. Another study reported the incidence of delirium to be 22.9% [30].

The results from these studies are significantly lower than those reported in international studies, which range from 45% to 87% [31, 32]. Whether this is solely due to sampling error or genuine distinctions between the populations studied, such as demographics, mixed ethnicities, or comorbidities needs to be determined through more extensive studies in Pakistan. As a result, the aims of this initial multicenter cross-sectional study of delirium in Pakistan ICUs were to (1) establish the point prevalence of ICU delirium among a cohort of critically ill patients in Pakistan and (2) identify the risk factors linked with the development of delirium. Understanding the prevalence and predictors of delirium is crucial for healthcare providers to implement appropriate preventive and management strategies. This study conducted in Pakistan aimed to determine the prevalence of delirium and identify associated risk factors in critically ill patients across various intensive care units (ICUs) in the country. Delirium is known to be influenced by factors such as age, severity of illness, and mechanical ventilation. However, there is a scarcity of data on delirium in Pakistani ICUs. Therefore, this study fills a critical knowledge gap and provides insights into the prevalence, predictors, and associated factors of delirium in critically ill patients, enabling healthcare providers to optimize patient care and outcomes in this population.

## Materials and methods

Study design

The Raosoft sample size calculator (Raosoft Inc., Seattle, WA) was used to calculate a representative sample size of 377 patients [33]. A confidence level of 95% was considered significant and the margin of error was assumed to be 5%. We conducted a one-day cross-sectional multicenter study on the 16th of April, 2023 in eight randomly selected ICUs across seven randomly selected tertiary care teaching hospitals in Pakistan using the convenient sampling method. The hospitals included Maroof International Hospital, Khyber Teaching Hospital, Lady Reading Hospital, Hayatabad Medical Complex, Nishtar Hospital, Services Hospital, and Allama Iqbal Memorial Hospital. The size of the units ranged between 30 and 50 ICU beds. In total, 35 tertiary care hospitals were reached out to and the hospitals were selected after seeking an expression of interest and no incentives were provided to the participants. Only seven hospitals were finally selected and the rest of the hospitals declined inclusion in the study. To augment the representation of large tertiary care hospitals, secondary and private hospitals were not included. Geographical representation was also considered. We included a convenience sample of hospitals located in two major provinces of Pakistan as well as the federal capital district. All the ICUs are closed, multidisciplinary units, and have well-established pain and sedation protocols. On April 16, 2023, eligible patients aged 18 years or more who were expected to stay in the ICU for at least 24 hours and had a RASS score ≥− 3 were screened for possible enrollment. We excluded patients admitted to the ICU following a traumatic brain injury, those with documented dementia in their medical chart as defined by the patient's primary care physician/psychiatrist, and those who were unable to participate in a valid delirium assessment (e.g., ICU admission because of acute or chronic neurologic disease and/or severe electrolyte disturbance). The ICDSC [17] was used as the delirium screening tool. While the hours of assessment were not standardized across sites, trained ICU physicians and/or critical care clinical pharmacists who were part of the research study completed the scale in the morning. The evaluations utilized included information from the last 24 hours. Delirious patients were defined as those with an ICDSC score of ≥4 [17]. We used the Strengthening the Reporting of Observational Studies in Epidemiology (STROBE) statement to draft this manuscript [31].

The study was conducted in accordance with the Declaration of Helsinki [34] and the International Council for Harmonization-Good Clinical Practice. It was reviewed and approved by the Institutional Review Board of Khyber Medical College.

Data collection and management

Study data were collected and managed using Research Electronic Data Capture (REDCap, Vanderbilt University, Nashville, TN) electronic data capture tools hosted at Khyber Teaching Hospital, Peshawar, Pakistan. REDCap is a secure, web-based software platform designed to support data capture for research studies, providing (1) an intuitive interface for validated data capture; (2) audit trails for tracking data manipulation and export procedures; (3) automated export procedures for seamless data downloads to common statistical packages; and (4) procedures for data integration and interoperability with external sources [32, 35]. Investigators for each study site underwent training on data entry and management, and each facility was granted independent access using user-authorized credentials. The sharing of protected health information was not permitted between sites, and hard copies of the data collection forms were made available to all study investigators as necessary. Site investigators were instructed on how to use the ICDSC through online educational materials on delirium, and an independent delirium expert was on hand to provide assistance. Prior to the commencement of the study, a conference call was arranged to discuss the application and potential pitfalls of the ICDSC and REDCap electronic data capture system with all investigators.

Data analysis

The IBM SPSS Statistics for Windows, v.24 (IBM Corp., Armonk, NY) software was utilized for the statistical analysis. To describe the study sample and test hypotheses, both descriptive and inferential statistics were employed. Means and standard deviations were calculated for normally distributed data, and medians with inter-quartile range were used for nonparametric data to present descriptive results, including graphical displays for all quantitative/continuous variables. Qualitative categorical variables were described using frequencies and percentages. Bivariate analysis was performed to compare quantitative/continuous variables using either the independent sample t-test or Mann-Whitney U-test as appropriate, while Pearson chi-square analysis or Fisher's exact test was used to compare all qualitative categorical variables between patients with and without delirium. To identify significant independent factors associated with the presence of delirium among patients, a multiple logistic regression model was generated and tested after adjusting for potentially confounding factors. The significance of each predictor was determined by computing the Wald test. Adjusted odds ratios and 95% confidence intervals for the adjusted odds ratio were reported. A two-tailed p-value less than 0.05 was considered statistically significant.

## Results

**Figure 1:**
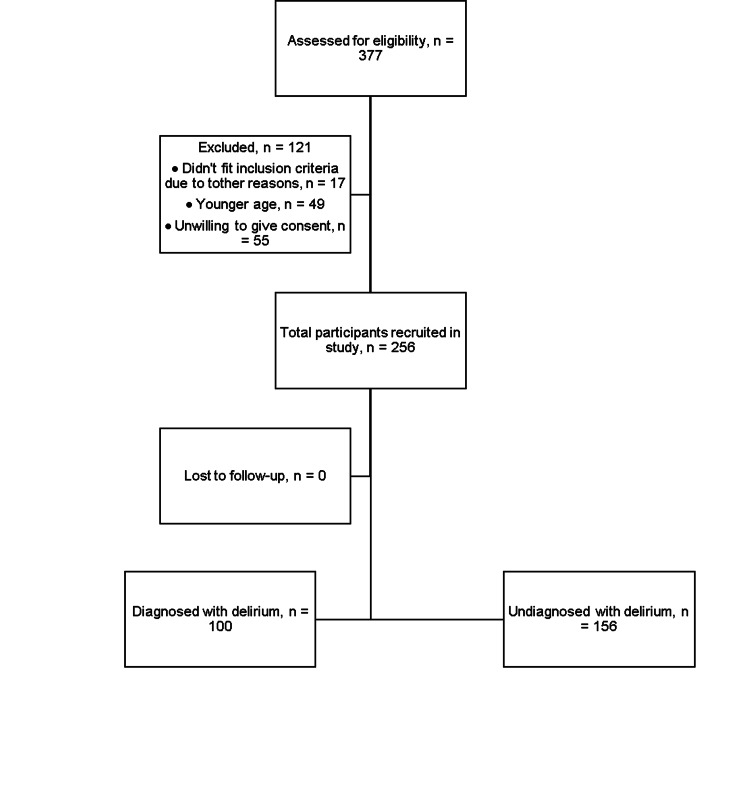
Flowchart of study participants

**Figure 2:**
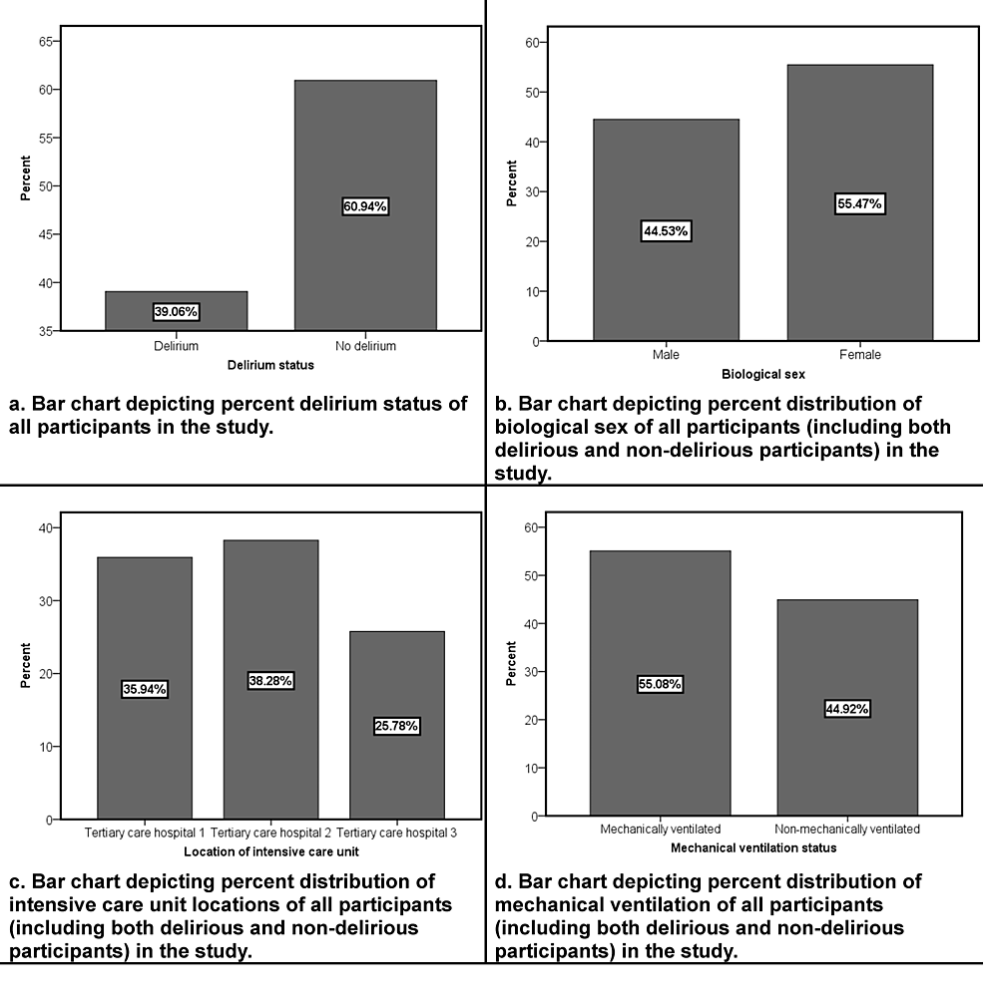
Bar charts representing the percent distributions of delirium status, biological sex, location of intensive care units and mechanical ventilation status of all study participants.

**Figure 3:**
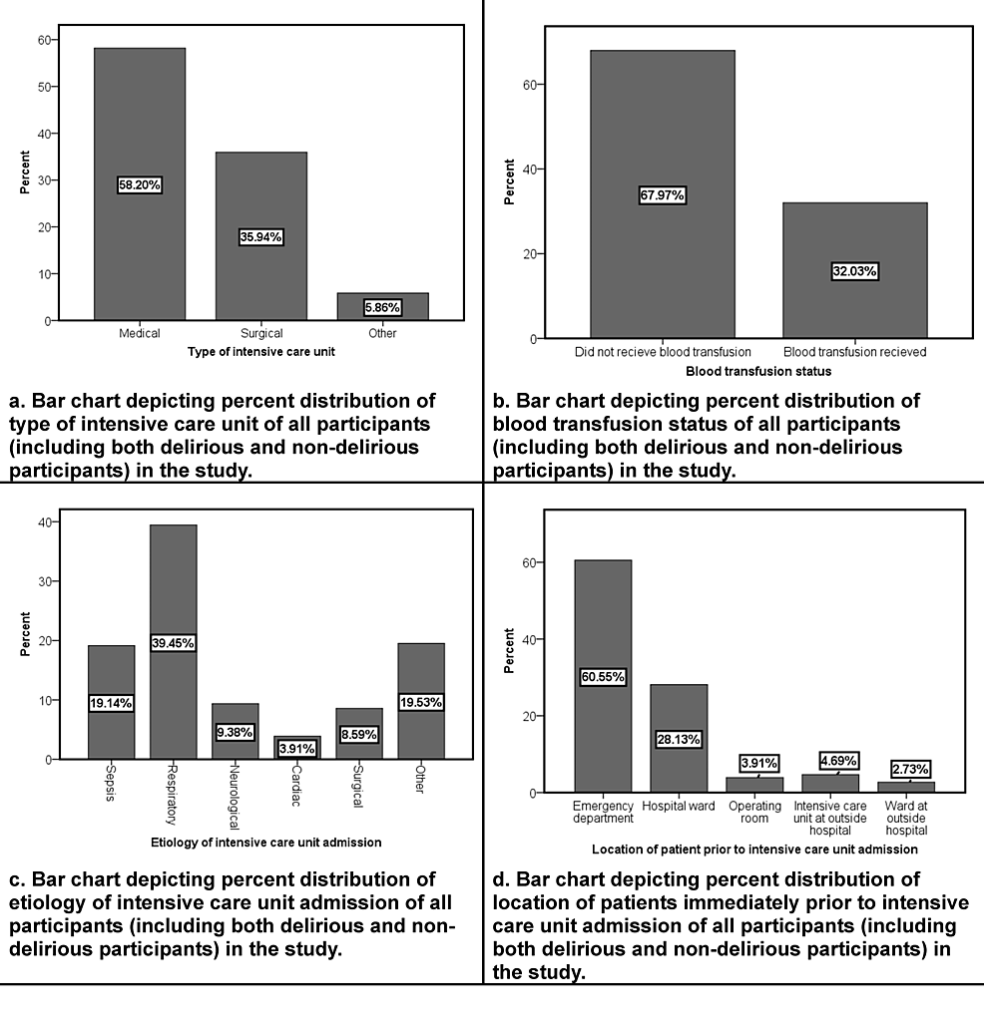
Bar charts representing percent distributions of types of intensive care units, blood transfusion status, etiology of intensive care unit admission and location of patient prior to intensive care unit admission of all study participants.

The study involved a total of eight ICUs, with four from the Punjab province, three from the Khyber Pakhtunkhwa province, and one from the federal capital region of Pakistan. Out of the 377 patients who were screened on the day of the study, 121 were excluded. The primary reason for exclusion was a low level of consciousness (defined as a RASS of less than -3), which was observed in 82 of the screened patients. Details regarding the interpretation of this scale are available at mdcalc.com [36]. The remaining 256 patients who met inclusion criteria were evaluated for delirium, and the overall prevalence of delirium was found to be 39.0%. The majority of patients were admitted to the intensive care unit because of respiratory disease. A detailed description of the reasons for ICU admission for different patients is shown in Table 1. Figure 1 represents the visual flowchart of study participants. The flowchart helps visualize the text in a way that makes it easy for the reader to understand the process from stages of screening to the outcomes. Figures 2-3 show different variables visualized as bar charts. The three hospitals in Figure 2c named Tertiary Care Hospital 1, 2 and 3 respectively are an aggregate representation of total hospitals per region/province.

Table 1 provides an overview of the baseline characteristics of the study participants, grouped by their delirium status. Among those who experienced delirium, 49% were male and had an average age of 53.79±16.20 years. The delirious patients tended to be younger (P < 0.001) and have more severe illnesses as reflected by their APACHE-IV scores (P <0.001. Although not statistically significant, delirious patients required mechanical ventilation (P = 0.594) more compared to those who did not experience delirium. The delirious group had lower median RASS scores compared to the nondelirious group (P <0.001).

**Table 1:** Clinical characteristics of the study participants. *P value has been calculated using Pearson Chi-Square test, ‡P value has been calculated using independent sample t-test, †P value has been calculated using nonparametric Mann–Whitney U-test, SD: Standard deviation, RASS: Richmond Agitation–Sedation Scale, APACHE: Acute Physiology and Chronic Health Evaluation, ICU: intensive care unit, IQR: interquartile range.

Clinical characteristic	Delirium (n=100)	No delirium (n=156)	p-value
Age (years), mean ± SD	53.79±16.20	59.07±8.94	<0.001^‡^
Gender, n (%)			
Male	49 (49)	67 (43)	0.373*
Female	51 (51)	89 (57)	
RASS at enrollment, median (IQR)	−1 (−2–0)	0 (0–0)	<0.001^†^
APACHE IV, median (IQR)	51 (44–61)	40 (36–43)	<0.001^†^
Mechanically ventilation at time of assessment, n (%)			
Mechanically ventilated	52 (52)	67 (43.0)	0.594^†^
Nonmechanically ventilated	48 (48)	89 (57.0)	
Received blood transfusion (n)	39	41	0.029^†^
ICU type, n (%)			
Medical	74 (74)	75 (48)	
Surgical	18 (18)	74 (47)	
Other	8 (8)	7 (5)	
Etiology of ICU admission, n (%)			
Sepsis	25 (25)	24 (15.4)	
Respiratory	51 (51)	50 (32.1)	
Neurologic	12 (12)	12 (7.7)	
Cardiac	3 (3)	7 (4.5)	
Surgery	9 (9)	13 (8.3)	
Other	0 (0)	22 (32)	
Location immediately prior to ICU admission, n (%)			
Emergency department	54 (54)	101 (64.7)	
Hospital ward	34 (34)	44 (28.1)	
Operating room following surgery	3 (3)	5 (3.9)	
ICU at an outside hospital	5 (5)	5 (4.7)	
Ward at an outside hospital	4 (4)	1 (2.7)	

The prevalence of delirium was not influenced by other factors such as the admitting service, the type of ICU (medical, neuro, or surgical), the hospital ward or emergency room admission, the presence of sepsis upon admission, and blood transfusions.

After adjusting for confounding variables, multiple logistic regression analysis revealed that age, RASS at enrollment, APACHE-IV scores, mechanical ventilation at the time of assessment, and etiology of ICU admission were significantly associated with delirium (p < 0.05), while biological sex and blood transfusion status were not the independent predictors of delirium in critically ill patients as shown in Tables 2-3. Additionally, there was no noticeable independent effect of hospitals on the prevalence of delirium in critically ill patients.

**Table 2:** Model fitness metrics This table represents a concise synopsis of key statistical outcomes and model fitness metrics. These metrics encompass R-squared (0.906), Adjusted R-squared (0.903), Standard Error (0.152), F-statistic (340.957), and associated p-value (<0.001), collectively illuminating the model's efficacy and significance within the context of the observed data.

R	R-squared	Adjusted R-squared	Standard error of the estimate	Sum of squares	Degree of freedom	Mean square	F	p-value
0.952	0.906	0.903	0.152	55.202	7	7.886	340.957	<0.001

**Table 3:** Predictors of delirium in patients admitted to the intensive care unit. This table presents the adjusted odds ratios, 95% confidence intervals, and p-values for the predictors of delirium in ICU patients, as determined by a multiple logistic regression model. The seven predictors include age in years, biological sex, Richmond Agitation-Sedation Scale (RASS) at enrollment, Acute Physiology and Chronic Health Evaluation-IV (APACHE-IV) score, mechanical ventilation at the time of assessment, blood transfusion status, and etiology of intensive care unit admission. The table displays that age, RASS at enrollment, APACHE-IV scores, mechanical ventilation at the time of assessment, and etiology of ICU admission were significantly associated with delirium (p < 0.05), while biological sex and blood transfusion status were not. The adjusted odds ratio represents the change in the odds of delirium associated with a one-unit increase in the predictor, while controlling for all other predictors in the model. A p-value less than 0.05 indicates strong evidence that the predictor has an effect on the outcome.

Predictor	Adjusted OR (95% CI)	p-value
Age (years)	1.024 (1.020-1.028)	< .001
Biological sex (male vs. female)	0.997 (0.961-1.034)	0.886
Richmond Agitation-Sedation Scale (RASS) at enrollment	1.073 (1.037-1.110)	< .001
Acute Physiology and Chronic Health Evaluation -IV (APACHE-IV) score	0.952 (0.949-0.955)	< .001
Mechanical ventilation at time of assessment	1.247 (1.170-1.329)	< .001
Blood transfusion status	0.984 (0.921-1.051)	0.625
Etiology of intensive care unit admission	1.098 (1.078-1.119)	< .001

The exclusion of a subset of patients in our study, amounting to 121 individuals out of the initially screened 377 patients, did not significantly impact the representative sample size. These exclusions were primarily based on a low level of consciousness (RASS less than -3) observed in 82 patients. Although these exclusions were necessary to ensure accurate delirium assessment and maintain the validity of the study findings, our final sample consisted of 256 patients who met the inclusion criteria and were evaluated for delirium. The relevance of our results lies in the comprehensive evaluation of delirium prevalence and associated risk factors in a multicentric study involving eight ICUs across different regions of Pakistan. By analyzing a diverse patient population, we were able to establish a robust understanding of delirium in ICU settings and identify significant factors, such as age, RASS at enrollment, APACHE-IV scores, mechanical ventilation at the time of assessment, and etiology of ICU admission, that are associated with delirium development. These findings provide valuable insights for clinical practice, intervention strategies, and future research efforts focused on effectively managing delirium in critically ill patients.

## Discussion

The study in Pakistan aimed to determine the prevalence of delirium in critically ill patients in different ICUs across the country. Out of 377 screened patients, 121 were excluded, mostly due to low levels of consciousness. The remaining 256 patients were evaluated, and the overall prevalence of delirium was 39.0% (out of 256 included participants). The study found that several factors were significantly associated with delirium in critically ill patients, including age, RASS scores at enrollment, APACHE-IV scores, mechanical ventilation at the time of assessment, and etiology of ICU admission. However, biological sex and blood transfusion status were not independent predictors of delirium. The results were obtained using multiple logistic regression analysis, which revealed an R-squared value of 0.906, indicating that the model can explain 90.6% of the variability in the data. The F-test indicated that the model was statistically significant (p < 0.001), suggesting that the independent variables significantly predicted the presence of delirium in critically ill patients. A recently published review by Ormseth et al. [37] reported that several factors increase the risk of delirium. These factors include being older, having dementia or cognitive impairment, being frail, having a history of delirium or other central nervous system disorders, having multiple other medical conditions, using alcohol, experiencing depression, suffering from malnutrition, and having functional, visual, or hearing difficulties [37]. Although a majority of the participants who were diagnosed with delirium were above 50 years of age, we found a slightly higher point prevalence of delirium among younger participants. The unexpected finding that younger patients in our study developed delirium compared to older participants may be attributed to several factors. Firstly, the study population's overall health status and baseline cognitive function could differ between the age groups, with younger individuals potentially having more acute or severe illnesses that made them vulnerable to delirium. Secondly, the etiology of delirium can vary widely, and younger patients may have been exposed to specific triggers, such as substance abuse, infections, or medication side effects, that are more prevalent in their age group. In our study, the patients were put on mechanical ventilation and administered deep sedation due to several reasons. Firstly, respiratory failure was a prominent factor necessitating mechanical ventilation. The patients experienced compromised lung function caused by conditions such as acute respiratory distress syndrome (ARDS), pneumonia, chronic obstructive pulmonary disease (COPD) exacerbation, or severe asthma. Secondly, respiratory muscle fatigue was observed in some patients, requiring mechanical ventilation to alleviate the strain on their respiratory muscles and prevent respiratory failure. Additionally, patients diagnosed with acute respiratory distress syndrome (ARDS) received mechanical ventilation with specific parameters like low tidal volumes and positive end-expiratory pressure (PEEP) to enhance oxygenation and minimize further lung damage. Deep sedation was concurrently administered to ensure patient comfort, minimize agitation or resistance to the ventilator, and promote adequate rest. Lastly, mechanical ventilation and deep sedation were employed in situations where intubation was necessary to protect the airway, such as severe trauma, drug overdose, or impending respiratory failure.

Patients in the study were evaluated as per RASS criteria during the day for the presence of delirium due to resource constraints and the need to balance patient care priorities. Validated delirium assessment tools are designed to capture symptoms over a specified period, providing a snapshot of delirium status during that timeframe. Delirium in critically ill patients often fluctuates, and frequent evaluations could disrupt patient rest and sleep, potentially worsening their condition. Therefore, assessing patients once a day strikes a balance between resource utilization, capturing delirium status, and considering patient well-being.

In our study, several factors contributed to the lower incidence of delirium compared to data reported in the literature. Firstly, our study included a specific patient population admitted to ICUs, which may have had characteristics associated with a lower risk of delirium, such as a less severe illness. Additionally, the centers where our study was conducted implemented robust preventive measures aimed at reducing the occurrence of delirium. These measures encompassed early mobilization, optimized pain management, improved sleep patterns, minimized sedative use, and the creation of a patient-centered environment. We hypothesize these may have led to the lower prevalence of delirium reported in our study compared to the data in the literature.

Our study is the first multicenter research in Pakistan to investigate the prevalence of delirium in critically ill patients over a single day. According to our findings, the incidence of ICU delirium is quite high with a lower occurrence in mechanically ventilated patients. This result is not consistent with other studies on delirium in critically ill mechanically ventilated patients [4, 8]. On the other hand, previous meta-analyses reported a higher risk of delirium development in mechanically ventilated critically ill patients than those not receiving mechanical ventilation [38]. Many reasons have already been described before, but an additional reason for the inconsistency in findings could be the inadequate screening and diagnosis of delirium in ICU patients due to variations in the expertise and training of healthcare professionals involved in our study, as well as differences in the availability of resources in our study, compared to the studies in the literature [39]. Our results indicated that there was not much variation in delirium frequencies among the participating sites, implying that the prevalence of delirium is relatively consistent among different regions of the country. As we compare the findings of our study with the research articles we have reviewed, we observe some similarities and differences. The study by Ali et al. [28] conducted in a surgical intensive care unit (SICU) also reported that advanced age, prolonged mechanical ventilation, and sedative use were significant risk factors for delirium [28]. Similarly, Nasir et al. [30] reported that advanced age and prolonged hospital stay were significant risk factors for delirium and subsyndromal delirium in older adults [30]. However, our study had some distinct differences. Unlike the study by Ali et al. [28] that only included patients in a SICU, our study included patients from multiple ICUs. Earlier studies have reported that patients with delirium are typically over 65 years of age and have an APACHE II score of 20 or more [18, 40-44]. In contrast, the severity of illness among study populations has varied widely, and the age of patients with delirium has been reported to range between 64 and 70.7 years in other studies [45-47]. Our study's cohort showed a mean age of 56 years among patients with delirium, with a median APACHE IV score of 51.

Our study has several strengths, including its prospective multicenter design and the inclusion of a large number of patients from various ICUs across Pakistan. We used the ICDSC tool, a validated tool for delirium detection in the ICU, for delirium detection. Thus, we believe that our findings have high internal and external validity, increasing their generalizability.

However, our study also has a few limitations. One potential limitation is that the participating ICUs may not be representative of all ICUs. Additionally, delirium assessment occurred only once during the ICU stay, which may have missed some delirium episodes but this was inevitable so that patient discomfort could be minimized and resource utilization was ensured in an adequate manner in a resource-scarce environment. Furthermore, we did not collect data on delirium severity. Another limitation was that this study was a one-day point prevalence study and may be subject to potential seasonal selection bias. Finally, our results may have been affected by confounding variables such as baseline health status, ambient noise, and light exposure. The study highlighted the utilization of mechanical ventilation and deep sedation in the patient population, as well as the limitations of assessing delirium in critically ill patients, such as resource constraints and the need to balance patient care priorities. Furthermore, our study revealed a lower incidence of delirium compared to previous literature, potentially due to the characteristics of the patient population and the implementation of preventive measures. The findings provide valuable insights for healthcare providers, emphasizing the importance of identifying risk factors and implementing appropriate measures to prevent and manage delirium in critically ill patients.

Overall, the study provides valuable insights into the prevalence and predictors of delirium in critically ill patients in Pakistan. The findings suggest that healthcare providers should be aware of the risk factors for delirium and should take appropriate measures to prevent and manage it. To confirm the relationship between ICU delirium and individual risk factors and to describe delirium outcomes in critically ill patients in Pakistan, further evidence from randomized, prospective clinical trials is necessary.

## Conclusions

Significant associations were observed between delirium and several key factors, including age, RASS scores at enrollment, APACHE-IV scores, mechanical ventilation status at the time of assessment, and the underlying etiology of ICU admission. The high prevalence of delirium, observed at 39%, emphasizes the importance of proactive screening and effective management strategies in the ICU setting.
